# Tissue-Mimicking Material Fabrication and Properties for Multiparametric Ultrasound Phantoms: A Systematic Review

**DOI:** 10.3390/bioengineering11060620

**Published:** 2024-06-18

**Authors:** Adel Jawli, Wadhhah Aldehani, Ghulam Nabi, Zhihong Huang

**Affiliations:** 1 Division of Imaging Sciences and Technology, School of Medicine, Ninewells Hospital, University of Dundee, Dundee DD1 9SY, UK; 2 Department of Clinical Radiology, Sheikh Jaber Al-Ahmad Al-Sabah Hospital, Ministry of Health, Sulaibikhat 13001, Kuwait; 3 School of Science and Engineering, University of Dundee, Dundee DD1 4HN, UK

**Keywords:** phantom, tissue-mimicking materials, ultrasound, elastography, flow phantom

## Abstract

Medical imaging has allowed for significant advancements in the field of ultrasound procedures over the years. However, each imaging modality exhibits distinct limitations that differently affect their accuracy. It is imperative to ensure the quality of each modality to identify and eliminate these limitations. To achieve this, a tissue-mimicking material (TMM) phantom is utilised for validation. This study aims to perform a systematic analysis of tissue-mimicking materials used for creating ultrasound phantoms. We reviewed 234 studies on the use of TMM phantoms in ultrasound that were published from 2013 to 2023 from two research databases. Our focus was on studies that discussed TMMs’ properties and fabrication for ultrasound, elastography, and flow phantoms. The screening process led to the selection of 16 out of 234 studies to include in the analysis. The TMM ultrasound phantoms were categorised into three groups based on the solvent used; each group offers a broad range of physical properties. The water-based material most closely aligns with the properties of ultrasound. This study provides important information about the materials used for ultrasound phantoms. We also compared these materials to real human tissues and found that PVA matches most of the human tissues the best.

## 1. Introduction

Medical imaging has allowed for significant advancements in the field of ultrasound procedures over the years. It began with general B-mode images and progressed to general images with varying echogenicities. This development eventually led to colour and power Doppler ultrasound, which is used to detect cardiovascular abnormalities. Doppler ultrasound is also employed to diagnose other conditions such as cancer by examining microvessel accumulation [1]. On the other hand, contrast-enhanced ultrasound (CEUS) eliminates limitations and enhances diagnostic performance by scanning blood diffusion in abnormal tissue, which is dependent on physiological processing [2]. In the early 1990s, ultrasound technology produced colour images that indicated a tissue’s physical properties and the degree of displacement caused by compression induction, creating an elastography ultrasound image. However, the advancements did not stop there; the technology has since evolved to provide quantitative results of tissue stiffness based on shear wave velocity converted into Young’s modulus [3]. Each imaging modality possesses distinct limitations that can differently affect the accuracy. It is important to ensure the quality of each modality to identify and eliminate limitations, which is usually achieved by tissue-mimicking material (TMM) phantoms.

**Figure 1:**
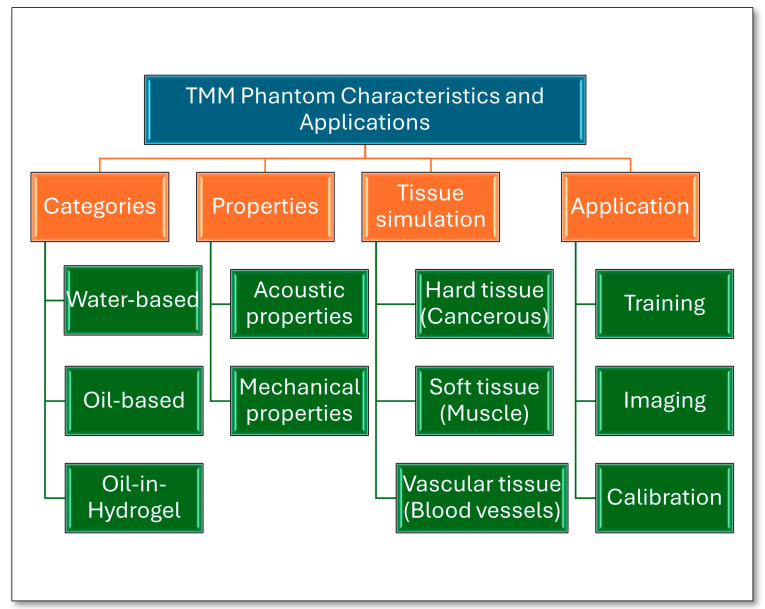
The characteristics and applications of TMM phantoms.

Tissue-mimicking material phantoms are models made to mimic human tissues’ physical properties. They offer an array of benefits for medical imaging quality assurance, study validation, and pre-training. Given the ethical considerations surrounding testing on human tissue, TMMs provide a valuable and ethical alternative for conducting essential research and clinical development [4,5]. These materials are either natural or synthetic polymer mixtures, with water, oil, or both as solvents. Thus, they can be classified into three types: water-based, oil-based, and oil-in-hydrogel TMMs. The characterisation and application of tissue-mimicking material phantoms are shown in Figure 1. The water-based materials used are usually agar, gelatine, polyvinyl alcohol (PVA), polyacrylamide (PAA), and polyvinyl chloride (PVC), while paraffin gel and copolymers like styrene–ethylene/butylene–styrene (SEBS) are oil-based materials. Oil-in-hydrogel materials are mixtures of agar or gelatine gel with safflower oil.

There are several studies that reviewed the fabrication of TMM phantoms. For example, Culjat et al. [6] reviewed studies on TMM fabrication for grayscale ultrasound phantoms. They discussed general and anatomical phantom-building techniques along with assessing the acoustic properties of each material. Cao et al. [7] provided an overview of the TMM properties needed for constructing an elastography ultrasound phantom and assessed the acoustic and mechanical properties. A systematic review of material fabrication for a perfusion phantom was provided by Kamphuis et al. [8]; the study contains details of phantom designs and material properties and discusses perfusion phantoms for computed tomography (CT) and magnetic resonance imaging (MRI) besides ultrasound. In contrast, Dakok et al. [9] reviewed articles that studied common carotid arteries for ultrasound phantoms and discussed wall and wall-less flow phantoms. In addition, they described the acoustic and mechanical properties of the materials utilised. However, the focus was on standard carotid artery phantoms instead of all flow phantoms. A comprehensive review by Armstrong et al. [10] described the TMM properties needed for ultrasound-guided needle intervention phantoms. They discussed the acoustic and mechanical properties of agar, gelatine, PVA, PVC, silicon, and SEBS reported in 24 studies.

This study aims to perform a systematic analysis of tissue-mimetic materials used in creating multiparametric ultrasound phantoms, taking into account several factors such as the fabrication process and physical properties of the materials. The goal is to present the measurable outcomes of TMM phantoms’ physical properties and compare them with those of various human tissues.

## 2. Materials and Methods

A systematic review of different ultrasound parametric phantoms was performed using PubMed and the Institute of Electrical and Electronics Engineers (IEEE) databases. The results were filtered to include all articles from January 2013 to the end of 2023. The primary search targeted studies that provided TMM properties and fabrication processing for ultrasound, elastography, and flow phantoms, including ultrasound images. This study was registered with PROSPERO for systematic reviews (PROSPERO Registration ID 502666). The results of the search were determined based on the title and abstract. The search terms included the following: [tissue-mimicking materials phantom [Title/Abstract]] AND [[ultrasound [Title/Abstract]] OR [flow phantom [Title/Abstract]]] OR [elastography [Title/Abstract]]. Notably, the search terms included all possible keywords related to ultrasound techniques, and the use of NOT was avoided to ensure that no relevant articles were missed.

Systematic review tools were used to remove duplicates and screen for eligible studies. The inclusion and exclusion criteria are presented in Table 1. The standard for these criteria is to provide TMM phantom fabrication processing with acoustic properties, such as speed of sound and an attenuation coefficient, and an ultrasound image of the phantom. Moreover, TMM phantom-based categories such as water-based, oil-based, and oil-in-hydrogel materials were considered. Studies that created a tissue-mimicking material phantom based on fabrication processes from other studies were excluded, as well as studies that provided a phantom created by 3D printing a commercial phantom, to avoid repetition and provide novelty.

## 3. Results

**Figure 2:**
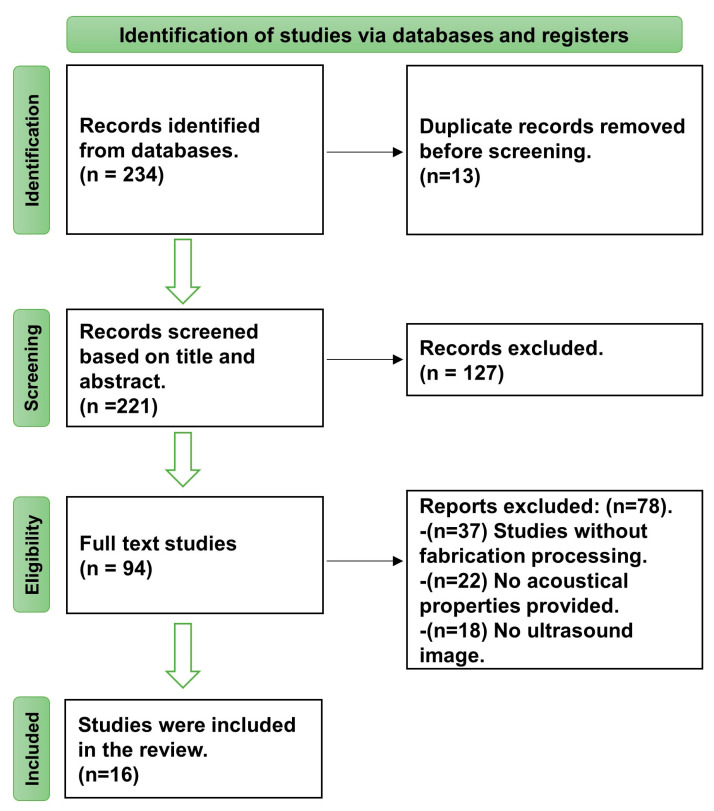
PRISMA flow diagram.

The identification, screening, and eligibility processes displayed in Figure 2 were instrumental in selecting the included studies, which totalled 234. Of those, 16 studies that were published between 2013 and 2023 were selected. The screening process involved various modalities such as CT, MRI, and PET/CT scans, and 75 studies were eliminated based on the criteria outlined in Table 1. Notably, studies on grayscale ultrasound phantoms had the highest representation compared to studies on other phantom modalities, such as elastography and flow phantom. Further, studies utilising water-based materials were higher than those that used oil-based and oil-in-hydrogel materials. The author’s name, year of publication, materials, phantom types, and study outcomes, such as the speed of sound, attenuation coefficient, and elasticity, are presented in Table 2. The outcomes of each study varied, and the details were carefully considered.

### 3.1. Grayscale Ultrasound Phantom

Water-based and oil-based materials have been used to create grayscale ultrasound phantoms. The following three studies focused on water-based materials, using agar based on International Electromechanical Commission (IEC) recipes: IEC agar [18], gelatine agar [19], and agar/wood [16]. Three studies employed PVA [15,17,20], while two others used PVC [14,21]. Meanwhile, three studies utilised SEBS and paraffin gel wax as oil-based materials [11,12,13]. IEC agar is the most recommended phantom material for simulating soft tissue, consisting of agar, glycerol, aluminium oxide (Al_2_O_3_), silicon carbide (Sic), benzalkonium chloride (BC), and water. The primary purpose of agar in the TMMs employed in phantoms is to replicate the physical properties of human tissue as closely as possible, whereas glycerol enhances the speed of sound and Al_2_O_3_ and (SiC) improve the attenuation coefficient and scattering, respectively. Additionally, benzalkonium chloride is often used to maintain the phantom’s stability [27,28].

A three-layered breast phantom designed for needle biopsy training was created by Ng et al. [18], in addition to an inclusion phantom that represents cancer tissue and is inserted inside the phantom’s layers. They studied the effects of various materials, including glycerol, Al_2_O_3_, olive oil, and surfactants, on the acoustic and mechanical properties of the standard IEC agar TMMs. They used both water-based and oil-in-hydrogel materials. The results indicated that adding SiC to the phantom improved backscatter, while Al_2_O_3_ had a negligible effect. Even when both were present, Al_2_O_3_ did not appear to reduce the backscatter enhancement associated with SiC. However, the B-mode ultrasound contrast was affected, potentially making the structures on the image less clear and distinct. Two water-based phantoms of IEC agar were created, one with a glycerol concentration of 120% and the other with a concentration of 110%, while both had the standard Al_2_O_3_ concentration of 260%. The speed of sound slightly increased in the first phantom, while the attenuation coefficient decreased in the second. The researchers found that adding olive oil and a surfactant reduced the acoustic properties and that glycerol reduced the attenuation coefficient. The results also showed that both the oil concentration and glycerol had a negative impact on the Young’s modulus. SiC was found to enhance the contrast of the inclusion phantom compared with the surrounding tissue, but the contrast was enhanced more by increasing the agar concentration.

Fohely et al. [19] mixed gelatine with agar in varying concentrations and added starch to create three distinct samples. In addition, they created two other samples, one without agar and the other without starch. The sample lacking agar exhibited the highest speed of sound, whereas the sample without starch had the lowest sound propagation. This suggests that adding starch improved sound speed, while agar’s presence reduced it. Conversely, agar improved the attenuation coefficient, with samples containing agar demonstrating higher attenuation coefficient values. Lastly, the researchers used an agar/gelatine mixture to create a homogenous kidney phantom under B-mode ultrasound.

Drakos et al. [16] created and tested a phantom using 2% *w*/*v* of granular agar and 4% *w*/*v* of wood powder. The purpose was to examine the impact of wood powder on the acoustic, nuclear magnetic resonance (NMR), and thermal properties. The results indicated that the attenuation coefficient was 0.53 dB/cm/MHZ with the addition of wood powder, surpassing the attenuation coefficient of the pure agar phantom without consideration of the temperature, which was close to 0.3 dB/cm/MHz [29]. However, the speed of sound did not increase with the addition of wood powder and remained in the range of 1487–1533 m/s depending on the temperature, which was lower than the speed of sound propagation in normal soft tissue.

PVA and agar are different in both fabrication and properties. PVA’s properties are influenced by various factors, including molecular weight (MW), freeze–thaw cycle (FTC) number, and PVA concentration. PVA was used for brain phantom fabrication by Elvira et al. in 2019 [15]. They added 10% *w*/*v* of high-molecular-weight PVA to 1,2-propanediol and Al_2_O_3_. The freeze–thaw cycle number was achieved through a three-step process involving freezing at −20 °C for 12 h, slowly thawing the phantom between freezing blocks, and then thawing at 5 °C for 2 h. The acoustic property was calculated after each FTC number until the fifth FTC was completed. The results showed that increasing the FTC number did not significantly affect sound speed and attenuation coefficients. The 10% PVA solution had a speed of sound of 1451 m/s and an attenuation coefficient of 0.15 dB/cm/MHz. When 5% *w*/*v* of 1,2 propanediol was added to the PVA phantom, the speed of sound increased by 1.7% while the attenuation coefficient remained stable. Furthermore, adding 5% and 10% of Al_2_O_3_ increased the attenuation coefficient to 0.55 and 0.9 dB/cm/MHz, respectively, and raised the acoustic impedance from 1.66 to 1.71 MegaRayl (MRayl).

Gautam et al. [17] used different concentrations of PVA to create a prostate phantom for transrectal ultrasound biopsy training. Three groups with varying concentrations of high MW PVA were subjected to eight FTCs, each of which involved freezing the phantom at −22 °C for 12 h and thawing it for 6 h at room temperature. The mechanical properties were tested after each FTC, while acoustic properties were only measured for four samples of PVA. The speed of sound and attenuation coefficient were recorded for 5%, 10%, and 15% PVA concentrations, highlighting the impact of PVA concentration on these properties. The elastic modulus ranged between 2.04 and 41.74 kPa, 8.31 and 113.20 kPa, and 32.53 and 130.57 kPa for the respective groups. Impressively, the elastic modulus increased until the sixth FTC before stabilising. An increase in the number of FTCs led to a change in the size of the PVA samples, particularly with the 15% PVA. The ultrasound image displayed a comparable level of brightness to the prostate image, even though PVA resulted in low backscatter.

Braunstein et al. [20] created a phantom made of PVA and cellulose powder to replicate kidney, liver, and spleen tissues. The phantom consisted of PVA with three different molecular weights: low, medium, and high. The FTC was repeated three times, with 5 h of freezing at −20 °C and 19 h of thawing at room temperature each time. The study found that there was no significant difference in the speed of sound and attenuation coefficient based on the MW of the PVA. In the process of fabricating the phantom, the low-MW PVA solution displayed low viscosity and low-temperature resistance. Meanwhile, a high-MW PVA phantom exhibited increased viscosity, leading to increased air bubbles. The PVA concentration had a linear relationship with acoustic properties. Furthermore, the cellulose powder played a vital role in increasing the attenuation coefficient.

Matheo et al. [14] studied the properties of polyvinyl chloride plastisol (PVCP) to create breast phantom tissues. Three samples of PVCP were tested, with each sample mixed with a different material such as a hardener (1:1), DOP (dioctyl phthalate) with graphite, or Al_2_O_3_. The results showed that PVCP with and without the other materials had a speed of sound that was close to 1400 m/s for the three samples. The addition of graphite or Al_2_O_3_ to the PVCP did not significantly impact the attenuation coefficient. The ultrasound B-mode of the fabricated phantom was very similar to that of an actual patient breast phantom, except for the glandular tissue, which was made from PVCP and Al_2_O_3_. This can be attributed to the backscatter.

Hariyanto et al. [21] used PVC, DOP, and graphite silicon oil to examine the ultrasound acoustic properties and X-ray attenuation for breast simulation. The results showed that, as the PVC concentration increased, the speed of sound typically increased. However, increasing the quantity of PVC with DOP concentration may have also contributed to the increase in the speed of sound. Graphite had a significant impact on the density of the phantom, which in turn affected the speed of sound, while silicon oil had the opposite effect. The range of sound speed for the different concentrations of PVC with different materials was 1536.6–2021.2 m/s, which is comparable to that of human soft tissue. Furthermore, the study found that PVC concentration had a greater linear influence on attenuation than graphite and silicon oil.

Vieira et al. [11] used an oil-based breast phantom that comprised two different densities of paraffin-gel wax: medium-density type (MDT) and high-density type (HDT). This phantom was created with 4% *w*/*w* of glass microspheres for backscatter and carnauba wax to improve contrast. The goal of the study was to create diverse types of breast lesions by changing the concentrations of MDT and HDT materials. The phantom’s speed of sound and attenuation coefficient were measured within the ranges of 1440–1480 m/s and 0.32–2.04 dB/cm/MHz, respectively. The higher density of the HDT material resulted in a higher speed of sound than the MDT material. When the carnauba wax content increased, the ultrasound speed increased further but the attenuation coefficient decreased.

Glycerol is a substance that can be used to enhance the speed of sound in materials. Grillo et al. [12] used glycerol mixed with SEBS and TiO_2_ in a constant concentration to increase the speed of sound. The results showed that adding glycerol increased sound speed to 1487 m/s, although this was still lower than the average sound speed in soft tissue. Cabrelli et al. [13] created three groups by varying the concentrations of SEBS with low and high viscosity and adding a wide range of glycerol percentages. The first two groups examined the impact of glycerol on the speed of sound, while the third group measured the effect of changing the SEBS concentration. The study found that, while glycerol did increase the speed of sound linearly, it remained lower than the average sound speed in soft tissue. Additionally, a higher oil viscosity was associated with a higher speed of sound due to density. The SEBS concentration did not have a significant impact on sound propagation. However, both glycerol and SEBS can increase acoustic impedance, with glycerol having a more notable effect on the impedance.

### 3.2. Elastography Phantoms

**Figure 3:**
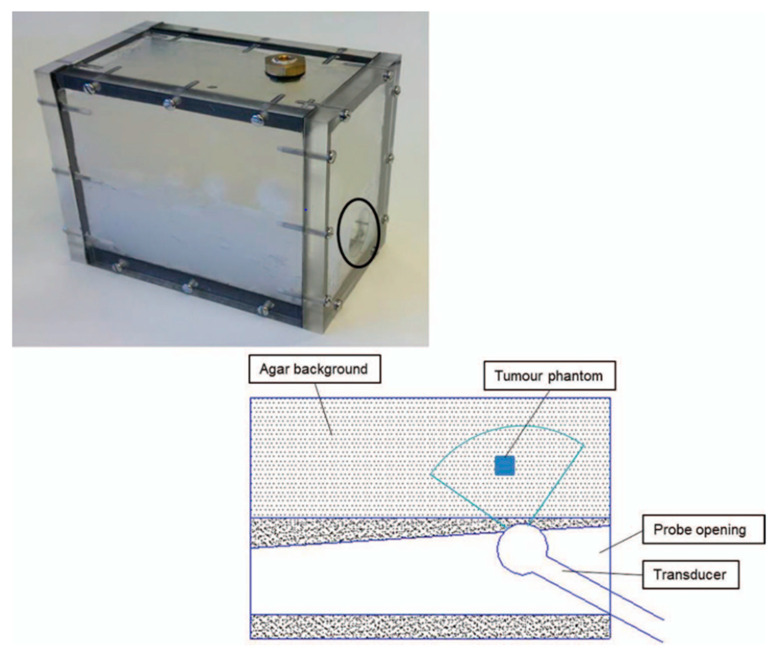
Tissue-mimicking prostate phantom (the black circle indicates the opening for the ultrasound transrectal probe). The diagram shows the placement of TMMs in the agar background at different levels in relation to the imaging transrectal probe [22].

**Figure 4:**
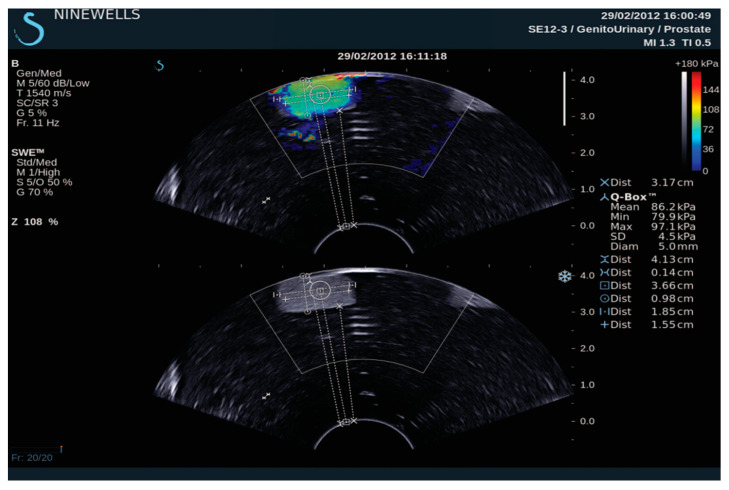
Quantitative estimation of Young’s modulus in the TMM phantom using the Supersonic Imagine Aixplorer system [22].

Two separate studies focused on the production of an elastography phantom. One study created a water-based material phantom [22], while the other employed an oil-based material [23]. In 2013, Cao et al. [22] created a prostate phantom using polyacrylamide (PAA) and combined it with varying concentrations of Al_2_O_3_ (Figure 3). They also analysed the mechanical and acoustic properties of agar with PAA and silicon; Al_2_O_3_ was added to agar and PAA only. The research demonstrated that an increase in the agar percentage generally improves the acoustic properties. Pure PAA produces a phantom with a low speed of sound and attenuation coefficient compared to the PAA with Al_2_O_3_. Silicon materials display a high attenuation coefficient and a very low speed of sound. Cao et al. found that a low concentration of agar can provide high elasticity; the Young’s modulus range for a 2–5% agar material was 157–443 kPa. Although PAA has a low Young’s modulus, it is in a high range compared to agar. In contrast, silicon had an elasticity value similar to that of agar. Cao et al. evaluated elasticity using an indentation machine and SWE ultrasound (Figure 4); the compression machine produced higher elasticity than the SWE-US. They found that the depth can have an adverse effect on the SWE result. Under B-mode and SWE ultrasound, PAA inclusion in the agar background yielded an image with acceptable contrast.

Oil-in-hydrogel phantoms are a widely accepted medium for validating viscoelasticity. Such materials exhibit both viscous and elastic behaviour. Nguyen et al., in 2014 [23], developed a phantom using various mixtures of gelatine, castor oil, graphite, and n-propanol. Their findings demonstrated that changing the percentages of gelatine and castor oil significantly impacted the speed of sound and attenuation coefficient. A small gelatine concentration resulted in a high speed of sound, matching soft tissue’s properties. However, an increase in gelatine led to a decrease in the attenuation coefficient. Castor oil, on the other hand, decreased both the speed of sound and the attenuation coefficient while enhancing the shear modulus and shear speed. An increased gelatine concentration increased the shear modulus. In contrast, the shear velocity was stable. However, 20% castor oil increased the shear velocity of the gelatine. In addition, the castor oil regulated the brightness of the B-mode under ultrasound.

### 3.3. Flow Phantom

The variation in flow phantom ultrasound lies in their respective designs. Two types of flow phantoms are commonly used—wall-less [24,25] and wall flow phantoms [24,26]. Wall-less phantoms use a tissue-mimicking material shaped to mimic the vessel lumen without an actual wall [30], while wall flow phantoms replicate the entire vessel structure with a physical wall.

Wang et al. [25] developed three innovative phantoms without walls, utilising gelatine-based, polydimethylsiloxane (PDMS)-based, and IEC materials. These phantoms were created to simulate larger blood vessels, validate microbubble studies, and improve special resolution, respectively. The phantoms’ speed of sound and attenuation coefficient were calculated to be 1554 m/s and 0.45 dB/cm/MHz for the gelatine-based phantom, 981 m/s and 4 dB/cm/MHz for the PDMS-based phantom, and 1541 m/s and 0.5 dB/cm/MHz for the IEC phantom. Interestingly, the PDMS-based phantom showcased better performance in terms of the ultrasound image clarity of microbubbles due to its increased transparency compared to the gelatine-based phantom.

Funamoto et al. [24] fabricated another wall-less phantom from different concentrations of PVA, dimethyl sulfoxide (DMSO), and glass microbeads. All samples of PVA underwent one FTC. The results revealed that increasing the PVA concentration did not affect the speed of sound but did increase the attenuation coefficient. This phenomenon also occurred when the DSMO concentration was increased while maintaining a constant PVA concentration. However, increasing the DMSO by more than 50% resulted in lower sound velocity and an increased attenuation coefficient. Two flow phantoms were generated and compared to the mouse carotid artery. Both phantoms had similar PVA and DMSO concentrations, but the second phantom lacked microbeads. Ultrasound imaging demonstrated that phantoms containing microbeads were comparable to in vivo vessels.

An oil-based phantom was used by Maneas et al. [26]. Varying concentrations of paraffin wax and glass spheres were added to a gel wax. The gel’s speed of sound was 1440 m/s, but with the addition of paraffin wax, it increased to 1448 m/s, and the average was 1.8 dB/cm/Mhz. The ultrasound image revealed that the paraffin gel increased the ultrasound brightness. Furthermore, they crafted cylindrical vessels and nerve phantoms with hypoechogenicity and hyperechogenicity ultrasound appearances, respectively. This was due to the vessel phantom without paraffin gel or a glass sphere. Additionally, the placenta phantom was created using pure gel wax and displayed hypoechoic centres and hyperechoic walls. For the heart phantom, gel wax and a glass sphere were used, and the heart chambers were filled with water instead of blood-mimicking material (BMM). The ultrasound showed a hemogenic phantom, while the heart chamber appeared slightly more hypoechoic.

### 3.4. Human Tissue’s Physical Properties

Ultrasound is a widely used technique for measuring the acoustic properties of materials, especially in clinical applications. It is possible to estimate ultrasound velocity and attenuation coefficients through this method. To measure the speed of sound, also known as sound velocity, the time-to-flight (TOF) method is commonly used [31], as reported by Duck et al. [32]. The speed of sound is calculated based on the time between the transmitted and received pulse in the medium, while the attenuation coefficient occurs due to sound energy loss. The attenuation is influenced by the medium’s sound absorption, reflection, and refraction and is proportional to the sound frequency. Table 3 and Table 4 display the sound speed, attenuation coefficients, and mechanical properties of both normal and abnormal biological tissues, as well as the tissue-mimicking material, respectively. The mechanical property of an elastography phantom is considered. If the phantom’s elasticity does not align with that of living tissue, the data collected may not be precise. Research suggests that mechanical testing and SWE-US are standard methods for measuring a phantom’s elasticity [33].

## 4. Discussion

This study aimed to investigate and comprehend the diverse tissue-mimicking materials applied in ultrasound, shear wave elastography, and flow phantom. As a result of this methodical review, investigators can distinguish between these materials based on their characteristics, types, and factors that affect their efficacy. Consequently, this study provided comprehensive data on each substance’s acoustical and mechanical features.

This review discussed water-based materials’ acoustical properties in comparison with human soft tissue, with agar and gelatine being particularly comparable. Agar has been the preferred material for TMM phantom fabrication since 1980 due to its higher melting point [54], although agar and gelatine exhibit similar acoustical properties. Agar boasts a wider range of Young’s modulus but requires additional enhancer materials to maintain the desired properties. A study [18] conducted using agar with varying concentrations of enhancer material yielded consistent results with those of [55]. The findings demonstrated that glycerol significantly increases the sound propagation velocity, but further increases in glycerol concentration will not lead to significant improvements due to the high speed of sound exhibited by glycerol compared to water. As a result, the sound speed increase caused by glycerol necessitates an increase in phantom density and bulk modulus. While Cannon et al. [55] showed that glycerol did not impact the phantom density, they agreed that the Young’s modulus is determined by glycerol and agar. IEC agar is commonly used in flow phantoms [25] to evaluate the spatial resolution in soft surrounding tissue [56]. However, the use of an IEC agar-based phantom in a wall-less phantom has limitations despite its superior properties, so it is often replaced with another material such as konjac or carrageenan. Therefore, while each material’s properties are significant in TMM phantom fabrication, it is equally important to review their limitations. IEC agar has acoustic properties that make it compatible with soft tissue. However, increasing the glycerol increased the elasticity [13].

The attenuation coefficient of the tissue-mimicking material is a critical factor in ultrasound imaging for achieving optimal brightness and preventing artefacts. In addition to Al_2_O_3_, TiO_2_ and BaTiO_3_ have been utilised. However, TiO_2_ is specifically employed to enhance optical scattering, while the limitation of BaTiO_3_ results in a reduction in the speed of sound [57]. Therefore, the careful selection of Al_2_O_3_ is essential for providing optimal acoustic properties.

PVA was studied in four studies, and, despite its complicated and time-consuming creation, it offers flexible properties. The most crucial factor to consider is the molecular weight. Both the molecular weight and the PVA concentration play a significant role in the polymer’s characteristics within the PVA phantom [58,59]. Consequently, one of the advantages of PVA is that a small amount of PVA with high MW can provide superior acoustic and mechanical properties. However, a low molecular weight of PVA requires high concentrations and FTCs to prevent damage during and after fabrication [20].

The second critical factor in PVA creation is the FTC process. The FTC process is the primary method used in PVA phantom fabrication and solidification. Studies show different results regarding the impact of the FTC process on the speed of sound [60,61,62]. It has been observed that the sound speed changes with increasing FTC numbers. This change is insignificant, indicating that the phantom’s size and shape, which alters with increasing FTC numbers, may be the primary cause of the change in sound speed [63]. PVA has a wide range of mechanical and acoustic properties suitable for several tissues, such as normal and abnormal prostate liver, breast, kidneys, blood vessel, and surrounding tissue in flow phantoms. However, PVA fabrication is a lengthy process that involves freezing and thawing, and its size and volume change with an increase in the FTC number.

Oil-based materials are identical to breast tissue in terms of physical properties but do not match the acoustical properties of several tissue types such as prostate, kidney, and blood vessels. However, oil is important in elastography phantoms to provide high shear wave dispersion [23]. This feature is essential for the accurate evaluation of tissue properties. However, many existing elastography phantoms are purely elastic and do not demonstrate dispersion, which limits their ability to fully mimic the behaviour of real tissues. Yet certain phantoms, such as those created to mimic liver tissue, are purposely crafted to display shear wave dispersion, therefore offering a more precise model for elastography research [64]. The speed of sound is typically related to the molecular structure, which can be affected by the temperature and bulk modulus. Moreover, the speed of sound can be affected by the density and bulk modulus according to SOS=√[B/ρ] (where *SOS* is the speed of sound, *B* is the bulk modulus, and *ρ* is the density). Oil has a lower bulk modulus and lower density. However, the speed of sound is also lower. Consequently, a small change in bulk modulus has a greater impact on the speed of sound than a comparable change in density [65]. This is corroborated by [55], which showed an increase in the speed of sound with stable density when increasing the glycerol concentration, and with [13], which demonstrated that both elasticity and speed of sound could be increased by increasing the glycerol concentration.

The present review was conducted with meticulous care using a systematic approach, but there were also certain limitations. Firstly, the number of studies reviewed was relatively small despite a large number of studies having been carried out on phantom fabrication between 2013 and 2023. This was largely due to a significant proportion of the ultrasound phantom fabrication studies failing to note the critical properties, including the speed of sound and attenuation coefficient [66,67,68]. Secondly, only one of the studies reviewed provided an SWE ultrasound image of the TMM phantom. Finally, the flow phantom studies failed to provide a Doppler image, either colour or power. Despite these limitations, the present review provides valuable insights into phantom fabrication, and future studies could address these limitations to further enhance our understanding of the topic.

The results of this systematic review suggest that future work should focus on developing and validating a multiparametric ultrasound phantom. This phantom can be created using polyvinyl alcohol (PVA). Further research is needed to understand why there is a change in the speed of sound due to an increase in the freeze-thaw cycle and to determine the benefits of using PVA to simulate soft tissue in flow phantoms instead of other materials, such as IEC agar, konjac, or carrageenan.

## 5. Conclusions

This systematic review examined studies that provide tissue-mimicking material properties for ultrasound, elastography, and flow. These properties are acoustic (speed of sound and attenuation coefficient) and mechanical (Young’s modulus). Studying the combination of these properties led to the creation of a multiparametric phantom to validate several ultrasound studies. PVA has physical properties that are identical to those of most normal and abnormal human tissues.

## Figures and Tables

**Table 1:** Inclusion and exclusion criteria.

Criteria	Inclusion	Exclusion
Setting	All countries	None
Modality	Ultrasound Doppler ultrasound Contrast-enhanced ultrasound Strain and shear wave elastography ultrasound	Studies that did not examine these devices
Outcomes	Acoustic properties Mechanical properties Fabrication processing Ultrasound image	Studies that did not detail the acoustic properties (speed of sound and attenuation coefficient) or TMM phantom fabrication processing
Study Type	TMM phantom fabrication	In vitro studies Review articles Systematic reviews 3D-printed phantom studies Commercial phantoms Studies that did not show new fabrication processing methods or depended on the fabrication methods of other studies
Publication Type	Journal articles	Conference abstracts, study protocols, reports, dissertations, books, or non-professional journals
Publication Year	Publication date in 2013 and after	Publication date before 2013
Language	English	All other languages

**Table 2:** Number of studies on each phantom type, the materials used, and the range of physical properties.

Modality	Author (Year)	TMMs	Phantom Type	SOS (m/s)	AC (dB/Mhz/cm)	Elasticity (kPa)
Grayscale	Vieira et al., 2013 [11]	Paraffin gel wax Glass microspheres	Breast phantom	1425.4–1480.3	0.32–2.04	
	Grillo et al., 2017 [12]	SEBS + glycerol + TiO_2_ + mineral oil	Photoacoustic phantom	1478.8	0.45	
	Cabrelli et al., 2017 [13]	SEBS + g + mineral oil		1423–1502	0.1–0.59	25.7–71.4
	Matheo et al., 2018 [14]	PVC plastisol + Al_2_O_3_	Breast phantom	1400	0.50	
	Elvira et al., 2019 [15]	PVA + propanediol + Al_2_O_3_	Brain phantom	1541–1622	0.15	
	Drakos et al., 2021 [16]	Agar and wood powder	US and MRI	1487–1533	0.48	
	Gautam et al., 2021 [17]	PVA	Prostate biopsy phantom	1478–1537	0.38–0.91	2–130
	Ng et al., 2022 [18]	IEC agar	Breast phantom	1479–1553	0.6–2	120–401
	Fohely et al., 2022 [19]	Gelatine + agar starch	Kidney phantom	1553–1956	0.31–0.47	
	Braunstein et al., 2022 [20]	PVA + cellulose	Kidney phantom	1556–1566	0.08–0.09	
	Hariyanto et al., 2023 [21]	PVC + DOP + graphite + silicon oil	Mammography and US phantom	1436–2021	0.51–0.063	
Elastography	Cao et al., 2013 [22]	PAA + agar + silicon + Al_2_O_3_	Prostate phantom	Agar: 1519–1534 PAA: 1468–1471 Silicon: 1187	Agar: 0.5–0.78 PAA: 0.26–0.70 Silicon: 4.09	Agar: 157.8–443 PAA: 104.3 Silicon 297.3
	Nguyen et al., 2014 [23]	Gelatine + castor oil + propanol + graphite	Visco-elastography phantom	1740–1558	0.4–1.07	2–11
Flow Phantom	Funamoto et al., 2015 [24]	PVA DMSO + glass microbeads	Ultrasound phantom	1567	40 (at 40 MHz)	
	Wang et al., 2017 [25]	Gelatine-based + PDMS	Flow pipe phantom	Gelatine-based: 1554 (PDMS): 981	Gelatine-based: 0.54 (PDMS): 4	
	Maneas et al., 2018 [26]	Oil + gel wax	Heart and placenta phantom	1443–1448	0.08–0.348	

TMMs = tissue-mimicking materials; SOS = speed of sound; AC = attenuation coefficient; US = ultrasound; MRI = magnetic resonance imagery; SEBS = styrene–ethylene/butylene–styrene; PVC = polyvinyl chloride; PVA = polyvinyl alcohol; PAA = polyacrylamide; IEC = International Electromechanical Commission; DOP = dioctyl terephthalate; DMSO = dimethylsulfoxide; and PDMS = polydimethylsiloxane.

**Table 3:** Quantitative properties of human tissue.

Human Tissue	Speed of Sound (m/s)	Attenuation Coefficient (dB/mm at MHz)	Young’s Modulus (kPa)
Breast (fat) [32,34,35]	1420–1479	0.05	9.24
Breast (glands) [32,34,35]	1553		11.28
Breast (carcinoma) [32,34,36]	1437–1550	0.3	44
Normal kidney [32,37,38]	1560	1.1	5–23.6
Renal mass [39,40]	1516–1783	0.9–10.2	8.9–31.80
Normal liver [32,41,42]	1577–1592	0.5	5.1–7.2
Fatty liver [32,43,44]	1522–1553	1.20–2.4	19.8
Tumour in the liver [43,44]	1555	-	86.4
Prostate [45,46,47,48]	1561–1614	0.78	36–42
Prostate cancer [46,49,50]	1584	1.42	75–131
Blood vessels [32,51,52]	1560–1660	0.13–0.16	72–134
Blood [32,53]	1590	0.21	

**Table 4:** Quantitative properties of tissue-mimicking material from review phantom studies.

Tissue-Mimicking Materials	Velocity (m/s)	Attenuation Coefficient (dB/cm at 1 MHz)	Young’s Modulus (kPa)
**Water-based materials**			
Agar phantom [18,22]	1519–1550	0.175–0.78	157–443
Agar/gelatine [19,25]	1553	0.47–0.54	
Agar/gelatine/starch [19]	1882	0.36	
Agar/wood [16]	1487–1530	0.56	
Gelatine/starch [19]	1956	0.31	
PVA phantom [15,17,20,24]	1478–1622	0.08–078	2–146
PAA phantom [22]	1468–1471	0.26–0.70	104
Silicon [22]	1187	4.09	297
PDMS [25]	981	4	
PVC [21]	1536–2021	0.5–0.8	
PVC plastisol [14]	1400	0.43–0.55	
**Oil-based materials**			
SEBS [12,13]	1478	0.35	
Paraffin gel [11]	1480	0.3–2	
Gel wax/paraffin wax [26]	1443–1447	0.08–0.348	
**Oil-in-hydrogel materials**			
IEC agar/olive oil and surfactant [18]	1479	0.085	120
Gelatine/castor oil [23]	1554–1558	0.4–1.07	10–26
PVC/silicon oil [21]	1536–1600	0.5	

## Data Availability

The data presented in this study are available within the article.
